# Optimized Sensorimotor Activation Enhances the Control of Goal‐Directed Aiming Mediated by Real‐Time Visuomotor Transformations

**DOI:** 10.1111/ejn.70335

**Published:** 2025-11-19

**Authors:** Roberto Panichi, Samuele Contemori, Jacqueline A. Sullivan, Andrea Calandra, Cristina V. Dieni, Andrea Biscarini

**Affiliations:** ^1^ Department of Medicine and Surgery, Section of Human Physiology and Biochemistry University of Perugia Perugia Italy; ^2^ Centre for Sensorimotor Performance, School of Human Movement and Nutrition Sciences The University of Queensland Brisbane Australia; ^3^ Department of Philosophy Western University London Ontario Canada; ^4^ Department of Medicine and Surgery, Section of Nuclear Medicine and Health Physics University of Perugia Perugia Italy; ^5^ Department of Neurobiology University of Alabama at Birmingham Birmingham Alabama USA

**Keywords:** goal‐directed movement, motor control, motor learning, muscle vibration, visuomotor coordination

## Abstract

Improving motor abilities may result from sensory–motor stimulations involving repetitive mechanical vibratory applications focused on muscles or tendons. These stimulations activate the proprioceptive pathway, critical for effective motion coordination. Optimized focal muscle vibration (o‐fmv) paradigms can enhance motor control of goal‐directed movements, potentially influencing the proprioceptive contribution needed for visual–proprioceptive integration during planning and execution stages of motion. We examined whether o‐fmv affects goal‐directed movements when visual information is available in real time or when it needs to be memorized. We applied the o‐fmv to the shoulder muscles in healthy participants to affect their proprioception. Then, we assessed immediate and 1‐week‐later effects on upper limb aiming toward visual targets. Movements were prepared with vision and executed either with real‐time or memorized visual information. O‐fmv improved mean speed, smoothness, and accuracy primarily when movements were performed with real‐time visual information. These improvements began immediately and continued to increase after 1 week. Minimal effects were observed when movements relied on memorized visual information. Therefore, o‐fmv produces lasting improvements in motor control of goal‐directed movements supported by real‐time visual information. Our findings suggest that o‐fmv may enhance the brain's processing of proprioceptive information used during motion planning and execution, potentially leading to long‐term changes. These effects might involve stream pathways that coordinate goal‐directed actions by integrating real‐time visual information with proprioceptive inputs.

## Graphical abstract

Optimized muscle focal vibration (o‐fmv) applied to the shoulder muscles enhances the control of upper limb goal‐directed movements. Using 3D motion analysis, we examined aiming movements supported by different visual–proprioceptive frameworks, depending on whether visual information was available in real time or retrieved from memory. O‐fmv improved accuracy, mean speed, and smoothness, especially with real‐time vision. These benefits appeared immediately and persisted for at least 1 week, suggesting durable changes in the sensorimotor network that integrates real‐time visual information with proprioceptive inputs to control aiming movements.

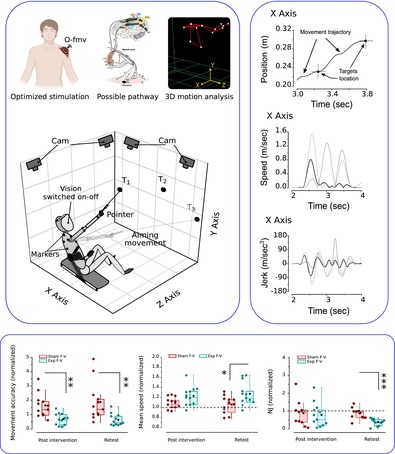

**Abbreviations**
AD: anterior deltoid muscle
ANOVA: analysis of variance
AVR: advanced virtual RISC (type of microcontroller)
DC: direct current (used in reference to the motor type)
EMG: electromyography
Exp: experimental group (received o‐fmv)
F‐V: full vision condition (real‐time visual information available)
Gpower: software used for power analysis
MVIC: maximal voluntary isometric contraction
NJ: normalized jerk
o‐fmv: optimized focal muscle vibration
PD: posterior deltoid muscle
r: effect size for nonparametric tests
SD: standard deviation
Sham: control group (received sham stimulation)
W‐V: without vision condition (visual information must be memorized)
ZigBee: wireless communication protocol
*ηp* ^2^
: partial eta‐squared (effect size in ANOVA)

## 1 Introduction

Goal‐directed movements are a prototype of coordinated human motion involving high‐level mental processing, such as aiming or reaching for a visible object with the hand (Battaglia‐Mayer 2019; Song and Nakayama 2009). Performing a movement directed at a target requires the brain to encode spatial information about the body and the target and generate dynamic phenomena, allowing the end‐effector to move into a designated space (Buneo et al. 2002; Biguer et al. 1982; Sober and Sabes 2005). Such features are standard in daily activities, athletics, and professional movements (e.g., reaching for food or tools for various scopes). Unsurprisingly, their damage is one of the major causes of disability resulting from neurological issues (Clarke et al. 2002; Kwakkel et al. 2003).

When vision and somatosensory information are available, the vision of the effector, target, background, and body proprioception conveys static and dynamic signals required to plan motion and transfer planning into goal‐directed movements (Redding and Wallace 1988; Sabes 2011; Ferraina et al. 2009; Goodman et al. 2018; Sarlegna et al. 2006; Rossetti et al. 1995). Additionally, environmental cues can be available to other sensory modalities, and it has been argued that optimal efficiency in sensorimotor coordination requires continuously updating the state of the task goal through afferent and efferent signals (Desmurget and Grafton 2000; De Comite et al. 2021; Scott 2016; Yeom et al. 2020).

However, although vision and proprioception can be combined as primary signals, their relative contributions may vary when encoding the location of the target and the end‐effector to define a spatial plan (movement vector), in transforming this plan into a motor command (inverse model) to generate effective motion, and during the online control of movement (Sober and Sabes 2005; Sarlegna and Sainburg 2009; Elliott et al. 2017; Ghez et al. 1991; Scheidt et al. 2005). How they are weighted is influenced by the sensory modality of the target (i.e., visual or somatosensory) and the reference frame in which computations for the motor command occur (Goettker et al. 2020; Monaco et al. 2010; Desmurget et al. 1998). When vision is available in real‐time and the target is in the visual modality, evaluating the spatial relationship between the target and the end‐effector (both within the visual framework) may rely primarily on vision (Sober and Sabes 2005; Monaco et al. 2010). At the same time, the computation of the motor command relies more on proprioceptive signals that need to be transformed into visual coordinates before being used, introducing errors due to coordinate transformations (Sober and Sabes 2005; Goettker et al. 2020; Gordon et al. 1995).

Nevertheless, goal‐directed movements toward a visual target remain possible without real‐time visual information, as in the case of internally driven actions, where proprioception may be combined with a temporarily memorized representation of the end‐effector and target location (Brunamonti et al. 2016; Land et al. 1999; Chieffi et al. 1999; Baddeley 2001; Heath and Westwood 2003; Heath 2005). It has been argued that movements executed with or without real‐time visual information rely on distinct visual–proprioceptive computations, depending on whether a direct visuomotor circuitry using online (real‐time) visual information or an indirect route using stored visual representations is engaged (Goodale et al. 2004; Milner et al. 2001; Westwood and Goodale 2003).

Extensive and specific practice can improve motor performance by optimizing movement coordination mechanisms (Winterbottom and Nilsen 2024; Khan et al. 1998; Scheidt and Ghez 2007). However, similar to findings in other sensorimotor domains, improving movement coordination may benefit from an alternative approach that does not require practice (Seitz and Dinse 2007; Contemori et al. 2020; Brunetti et al. 2006; Camerota et al. 2016; Toscano et al. 2019; Cordo et al. 2013; Fattorini et al. 2006; Attanasio et al. 2018). This approach may be most effective by promoting the activation of the proprioceptive pathway, possibly enhancing its resoluteness (Aman et al. 2014; Proske and Gandevia 2012; Filippi et al. 2023), to improve accuracy and kinematics (Sarlegna and Sainburg 2009; Gordon et al. 1995). This goal can be achieved through mechanical vibratory stimulation focused on the muscles' bellies or tendons (Manzo et al. 2023; Roll and Vedel 1982). Focal vibration exposes segmental proprioception to repetitive stimuli that may enhance spatial and temporal properties in the sensory input, potentially biasing the proprioceptive information processing from the stimulated area (DiZio et al. 1993; Verschueren et al. 1998; Fallon and Macefield 2007; Roll et al. 1989; Strzalkowski et al. 2017). Additionally, the enhanced sensory inflow caused by focal vibration may activate the targeted sensorimotor pathways and trigger adaptive changes, similar to what has been suggested for other repetitive stimulation (Steyvers et al. 2003; Rosenkranz and Rothwell 2012; Clapp et al. 2012; Amiez et al. 2024; O'Neill et al. 2019). It has been argued that these central effects may be involved in some of the immediate and enduring aftereffects observed in sensorimotor functions following vibration (Rocchi et al. 2018; Brunetti et al. 2012; Souron, Besson, McNeil, et al. 2017; Beste and Dinse 2013). For instance, vibrotactile stimulation applied to the skin area corresponding to an unseen somatosensory target has been shown to improve reaching accuracy toward that target (Mikula et al. 2018). Consistently, prolonged and repeated mechanical vibrations on upper limb muscles might ameliorate upper limb kinematics in goal‐directed movements of healthy performers and poststroke patients (Paoloni et al. 2014; Tavernese et al. 2013; Aprile et al. 2016; Zeng et al. 2023). We have previously developed an optimized stimulation paradigm of focal muscle vibration (o‐fmv) that can lastingly enhance sensory–motor transformation and improve coordination of the upper limb in target‐directed movements after application to neck or shoulder muscles (Contemori et al. 2020; Panichi et al. 2011; Pettorossi et al. 2015).

It remains uncertain which aspects of goal‐directed movement control are most influenced by vibration. We propose that focal muscle vibration may enhance proprioception, thereby improving visual–proprioceptive integration, which supports multiple stages of motor control (Sober and Sabes 2005; Goettker et al. 2020). Therefore, o‐fmv could facilitate not only movement planning but also the execution, potentially affecting accuracy and kinematics (Ghez et al. 1995; Mikula et al. 2018; Sarlegna and Sainburg 2009). To better situate these effects, we examined performance under different frameworks of visual–proprioceptive integration, based on visual information, whether available in real time or as memorized.

For this purpose, we applied the o‐fmv on muscles that assist with shoulder movements in healthy individuals to impact their proprioception. Then, we studied the o‐fmv aftereffect over time on upper limb target‐directed aiming, prepared in vision, and executed with real‐time or memorized visual information. We evaluated movement accuracy and kinematics in terms of speed and smoothness.

Our findings provide new insight into the mechanisms of muscle vibration and highlight its potential for regulating volitional motion coordination without task training.

## 2 Methods

### 2.1 Participants

For this double‐blind parallel‐group study design, 24 healthy volunteer performers (14 males and 10 females; mean age 30.6 years, range 22–41 years; mean height 175 cm, range 162–188 cm; mean weight 72 kg, range 51–93 kg; 21 right and three left‐hand dominant) were enrolled. The participants were randomly assigned to two groups: Group 1 (the experimental group, indicated as Exp; *n* = 12) underwent the o‐fmv, consisting of focal vibration over slightly and alternatively contracted anterior (AD) and posterior deltoid (PD) muscles of the dominant upper limb; Group 2, the control group (indicated as Sham group; *n* = 12), did not receive the o‐fmv; instead, they were administered a sham stimulus. Experimenters collecting and analyzing data were unaware of the treatment administered to the participants, identified by a randomly assigned code.

All participants were free from any upper limb and trunk motor deficits, and none were practicing a unilateral sports activity (e.g., tennis and volleyball) or competing at an elite athlete level in any sport for at least 12 months before and during the study. The participants were also free from cognitive, neurological, visual, and vestibular impairment and were not using any psychoactive drug or medication at the time.

All participants gave informed consent to their inclusion in the study, which was conducted following the Declaration of Helsinki (1964) and was approved by the local ethics committee of Perugia University.

### 2.2 Task Description

**FIGURE 1:**
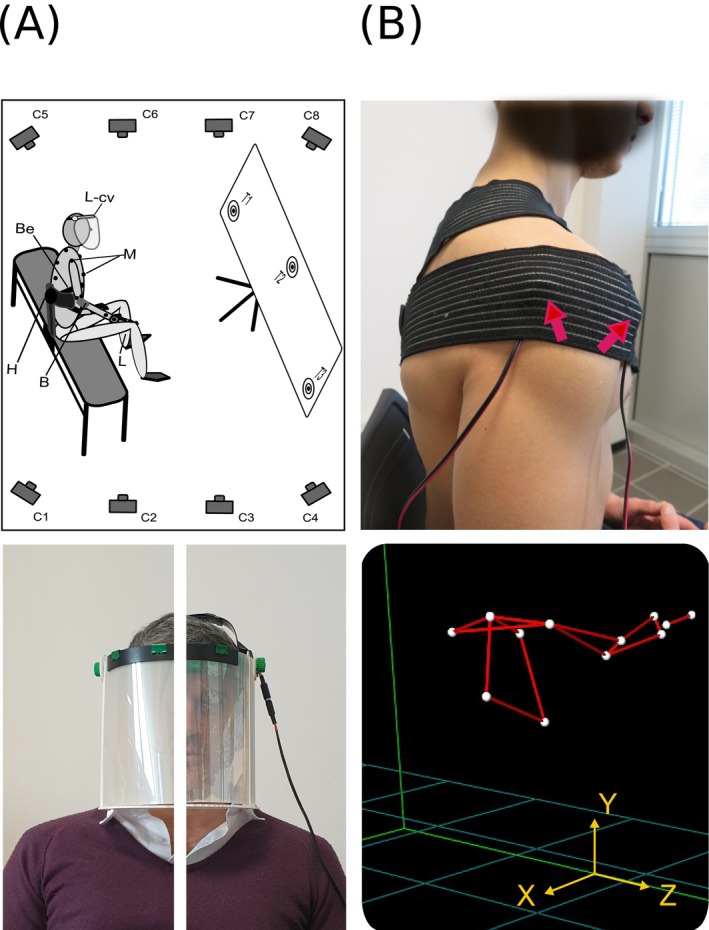
Experimental setup and stimulation. (A) Cartoon showing the experimental setup (top) and pictures of a subject wearing a crystal visor that could be turned remotely opaque or transparent (bottom) to switch off/on the visual field. Participants belonging to either the Sham or Experimental group (underwent o‐fmv) were seated, fastened on a bench, and used a laser pointer to perform randomly assigned targeted tasks. The tasks consisted of continuous aiming sequences at three targets positioned in front of the subjects and executed in either a visually guided (visor transparent) or internally guided condition (visor opaque). A 3D motion capture and analysis system comprising eight cameras and spherical reflective markers placed on subjects' upper limbs and trunks was used to analyze upper limb movement kinematics. (B) Picture shows the vibratory device application sites (top), while a 3D model of the upper limb is shown in the representation (bottom). Vibrators were placed over the anterior and posterior deltoid. B, bandage; Be, belt; C (1–8), cameras; H, holder; L, laser pointer; L‐cv, liquid crystal visor; M, markers; T (1–3), targets.

**FIGURE 2:**
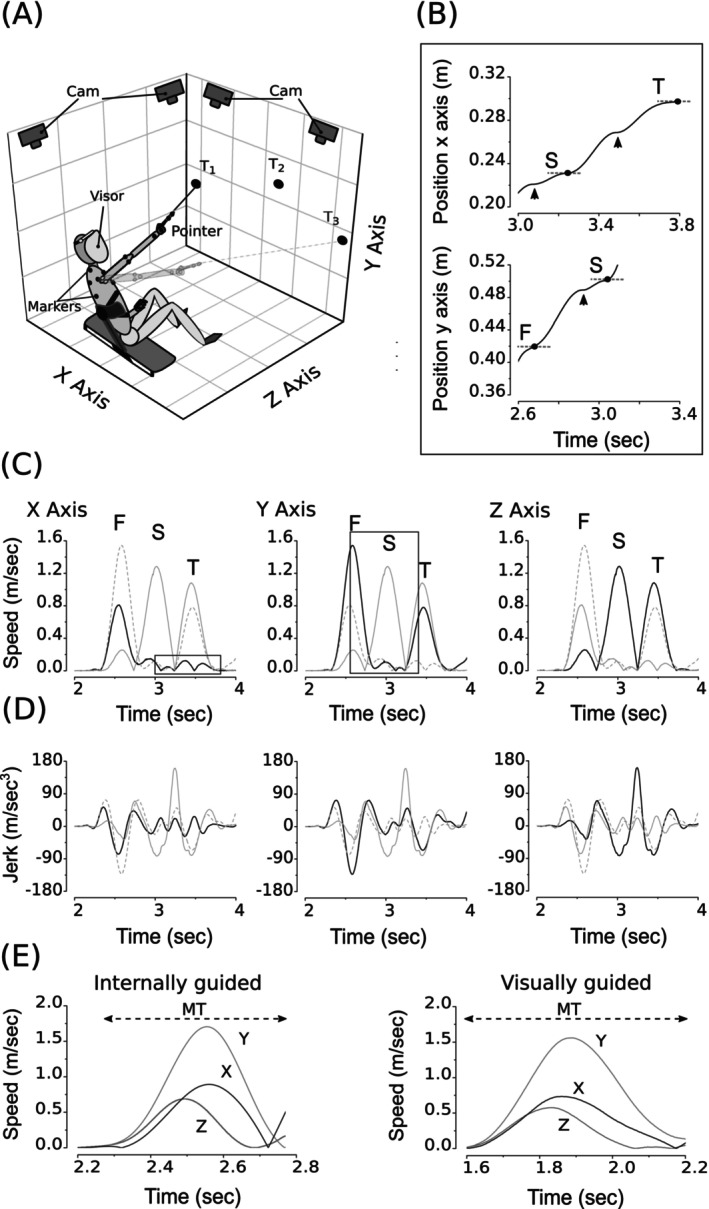
Motor task and movement analyses. (A) Cartoon represents a participant performing the internally guided aiming task (visor opaque). (B) Positional traces showing trajectories with visible inflections (arrows). F, S, T, first, second, and third target, respectively; T (1–3) targets. (C–D) Typical velocity (C) and jerk (D) traces of targeted movements. The speed signals within the frames refer to the positional traces shown in (B). Each graph shows traces on the corresponding plane (thick black trace) and the other 3D planes for comparison (thin and dashed gray traces). It is possible to notice multiple independent bell‐shaped profiles of velocity and multiple independent variations in jerk. (E) Example of 3D velocity traces of internally (left) and visually guided (right) movements. It shows a lower speed and longer movement time (MT), as well as a skewer profile for visually guided movement. Cam, cameras.

Participants had to perform a sequential, targeted task with their dominant upper limb without assistance while seated on a bench and outfitting a custom‐made crystal visor that could be remotely turned opaque to switch off their visual field (Figures 1, 2).

After sitting and being positioned, participants maintained a relaxed reference posture for about 2 min. This standardized settling period allowed posture and baseline muscle tone to stabilize, while experimenters verified marker visibility, visor status, and laser alignment. No rehearsal movements were performed during this interval.

The task required aiming a laser pointer at three targets (labeled 1 to 3) in two continuous sequences, 1‐3‐2 and 3‐1‐2 (Figures 1, 2) (Contemori et al. 2020). A 600‐Hz tone signaled the task's start, which subjects executed randomly with full vision (F‐V, visor transparent) or without vision (W‐V, visor opaque).

In the W‐V condition, during the 2‐min settling period, the visor was transparent, allowing participants to stabilize a reference of the initial hand, target, and background positions. The visor was then automatically turned opaque, followed by a 2‐s delay to verbal instructions on the aiming sequence to perform and a further 1‐s delay before the go tone. Thus, during motion preparation, real‐time visual and proprioceptive information may be integrated, whereas action decision, programming, and unfolding rely on proprioception and memorized visual information of the targets and limb position (i.e., memory‐guided action).

In the F‐V condition, the visor remained transparent throughout the 2‐min settling period. Participants then received the verbal sequence, and after 1 s, the go tone was delivered. Each movement phase was under visual control, and from planning to execution, participants relied on real‐time visual information and proprioception (i.e., visually guided action).

Participants were instructed to execute aiming sequences using the shoulder while extending the elbow, supported by a semirigid bandage and maintaining the wrist in a neutral position (Figure 2). The neutral wrist position was found individually, keeping the wrist in minimal flexion/extension and radial/ulnar deviation. Additionally, their trunk was fastened to a holder on the bench to stabilize their primary position during the task (Figures 1, 2).

The three targets were attached to a rectangular plywood support and arranged as the angles of an isosceles triangle (base 180 cm; height 60 cm), with target n.1 representing the left angle of the triangle and target n. 3 the right one (Figure 1). The rectangular plywood support was positioned as in Contemori et al. (2020) (Figure 1), aligned to the participants' frontal plane and placed at a distance that allowed the shoulder to move about 45° in the transversal plane, directing the pointer from target n.1 (n.3) to n.3 (n.1).

### 2.3 Procedure

#### 2.3.1 Maximal Voluntary Isometric Contraction (MVIC)

At the beginning of the testing session, each participant of both groups (Sham and Exp) performed a MVIC in manual resistance tests specific to AD and PD muscles of the dominant upper limb for force evaluation using a portable dynamometer and an electromyographic system with wireless probes placed on the muscles. Testing consisted of resisting forward flexion and backward extension at about 45° shoulder flexion. Two MVICs of 5 s were performed for each test, and a rest of 5 min was given between tests (Contemori et al. 2020). MVIC signals were used for offline normalization of the EMG and force data recordings (EMG analyses not reported in the present study). Additionally, the evaluation of MVIC was used to enable participants to maintain the intensity of the muscle contraction at a predetermined value (less than 10% of MVIC) during the intervention (Sham or o‐fmv).

#### 2.3.2 Calibration

After 10 min of recovery from the MVIC tests, participants were familiarized with the task following the examiner's instructions. Then, participants underwent a calibration phase in which they had to accurately aim the laser pointer and maintain the upper limb position at each target for about 3 s. During the calibration, the performers were seated on the bench, could see the targets, and could precisely aim at them, even with corrections. The calibration was made to determine the exact three‐dimensional position of the performers' dominant hand and arm when the pointer was “perfectly” on the targets. We used this information to estimate a parameter, which we refer to as movement accuracy, defined as the three‐dimensional spatial difference between the hand positions recorded during calibration and those recorded during the tasks.

#### 2.3.3 First Task Session and Basal Responses

Once the calibration phase was completed, each participant performed continuous aiming sequences (1‐3‐2, 3‐1‐2) either in the W‐V or F‐V condition, whose order was randomly assigned by the examiner. Starting from the “reference position” with the arm relaxed along the thorax and the hand placed over the ipsilateral thigh, participants had to execute the aiming sequences listed by the experimenter and return their upper limb to the reference position (Figures 1, 2). The reference‐start position was determined by requiring performers to locate their used hand in a predefined position on the ipsilateral thigh.

The assignment required executing the task as quickly and accurately as possible without any pauses in the designated movement sequence.

Participants were instructed to execute the aiming sequence, maintaining the same body posture and using an upper limb movement pattern as similar as possible to that used during the calibration phase (i.e., moving the shoulder while extending the elbow and maintaining the wrist in a neutral position).

#### 2.3.4 Intervention: The Optimized Vibratory Stimulation and Sham

After completing the first task, the Exp group underwent o‐fmv comprehending focal vibration of AD and PD of their dominant upper limb (Figure 1) (Contemori et al. 2020). In contrast, the Sham group was given a sham stimulus. The vibration was administered using two identical custom‐made devices with adjustable vibration frequency and amplitude. Each custom‐made vibrator consisted of a flat DC micromotor (Fritz Faulhaber GmbH & Co. KG, Schönaich, Germany) and an offset unbalanced mass rotating about the motor shaft with an output torque of 21 mN*m and a rotating speed capacity of up to 223 rpm. The micromotor and the mass were encased in a plastic case shaped like a cylinder (24 mm long and 26 mm across) perpendicularly to the cylinder axis. Vibrators were positioned to place the motor shaft parallel to the muscle fibers with the part that contacted the skin having a surface tip of 53‐mm^2^ surface tip. Vibration frequency and amplitude could be varied by changing the rotation speed through an electric motor's conventional proportional–integral–derivative control schema. The vibrator was controlled by a custom‐made AVR (Atmel Corp., San Jose, CA, USA) that communicated with a host personal computer via ZigBee wireless specification (Digi International Inc., Minnetonka, MN, USA). We used a mechanical vibration with 0.3–1‐mm peak‐to‐peak sinusoidal displacement at 100 Hz. The vibration displacement and frequency were calculated as described in Contemori et al. 2020.

The vibration was administered simultaneously to AD and PD muscles for the Exp group and was characterized by three subsequent administrations of 10 min, separated from each other by a resting period of about 2 min. To this end, the two vibrators were placed and fixed in a predetermined position by a Velcro band under an elastic bandage clinging to the muscles (Figure 1). The bandage maintained the vibrators over the muscles with force in the range of 190–220 g measured by a strain gauge (FlexiForce sensor model A401, Tekscan, Boston, MA, USA, width 31.8 mm, linearity error < 3%). Subjects were asked to contract AD and PD muscles slightly and alternatively during the vibrations. We used a portable dynamometer previously calibrated individually for each participant to maintain the contraction level under 10% (between 5% and 10%) of MVIC.

The AD contraction was executed by flexing, while the PD was executed by extending the shoulder against a fixed resistance coupled to the dynamometer. The AD and PD contractions were performed isometrically and alternately, maintaining the shoulder at about 45° of flexion, and lasted 2 min and 30 s each. Thus, the AD and PD received 30 min of vibration and were slightly contracted for 15 min each during the entire stimulation paradigm.

The stimulation we used might be optimal for an intense sensorimotor activation while minimizing the impact of the tonic vibration reflex (reflex caused by vibration) and contraction on fatigue induction that might impair motor performance (Fallon and Macefield 2007; Farabet et al. 2016; Ushiyama et al. 2005; Souron, Besson, Millet, et al. 2017; Casale et al. 2009).

Unlike the Exp group, the Sham group, representing the control, underwent “false” vibratory stimulation. The participants in this group were treated the same way as those in the Exp group, following identical procedures and using a device they believed was a vibrating device. They were informed that they were receiving vibratory stimulation, although no actual vibration was applied. However, they could hear a faint buzzing noise that resembled vibration.

#### 2.3.5 Second Task Session

Once the o‐fmv or Sham procedure was completed, to evaluate the post effects of the intervention (o‐fmv or Sham) on motor performance, each participant performed a second session of the targeted task, following the same procedures detailed for the first session.

#### 2.3.6 Retest

To evaluate the long‐term effects of o‐fmv (also referred to as late effects in the text), each participant underwent a retest session 1 week after treatment, during which we used the same procedures described for the first *task session*. Therefore, no o‐fmv or Sham was given again, and only the targeted task session was performed.

### 2.4 Kinematic Data Recording and Processing

The dominant upper limb 3D kinematics were recorded and analyzed with the use of a multicamera “Smart‐DX 6000” optoelectronic motion capture system, consisting of eight high‐resolution infrared cameras operating at a sampling frequency of 500 Hz (Figure 1) and “Smart Analyzer” software (BTS Bioengineering, Milano, Italy), respectively. This setup may introduce an intrinsic 3D error due to instrument calibration, with an average error of approximately 0.2–0.3 mm (SD = 0.25 mm).

Following the guidelines defined by the “shoulder sub‐committee” of the “International Society of Biomechanics Committee for Standardization and Terminology” (Wu et al. 2005), a set of 14 skin‐mounted spherical reflective markers, 5 mm in diameter, was attached to the following anatomical landmarks: the spinous processes of the seventh cervical vertebra and the eighth thoracic vertebra, the sternal notch, the xiphoid process, the lateral aspect of the acromion of both scapulae, the medial and lateral epicondyle of the elbow, the radial and ulnar styloid of the wrist, the anterior superior iliac spine of both iliac bones, and the second spinous processes of the sacrum, and the middle metacarpophalangeal joint of the hands, the latter used to calculate accuracy. The use of this set of markers, along with the recording and analysis systems, enabled a precise evaluation of the three‐dimensional position of the hand and arm during targeted movements and upper limb kinematics for offline determination of the spatial relationship between the wrist, elbow, and shoulder (Wu et al. 2005; Contemori and Biscarini 2018, 2019; Contemori et al. 2020). When aiming movements were executed differently from the way participants were instructed, the recordings were not used for evaluation of motor performance.

#### 2.4.1 Accuracy and Kinematic Indexes

To estimate the accuracy of the movement, we used the difference between the three‐dimensional spatial positions (mm) of the hand recorded at each target during calibration and those recorded during the tasks.

Moreover, we used the hand's three‐dimensional displacement to compute hand speed (m/s) during the task, as the first derivative of the hand positional signal, and determined the kinematics assessment peak and mean speeds. The onset and end of movements were defined using a velocity threshold of 0.02 m/s.

Besides, we calculated the third derivative of the three‐dimensional position signal of the hand (indicated as “J” in the text) to evaluate normalized jerk (NJ), a unit‐free–dependent variable that reflects the smoothness of movement (Teulings et al. 1997; Flash and Hogan 1985) as follows:

NJ=12∫titfJ2tT5A2dt

where *T* is the time necessary to perform a targeted movement (movement duration), *A* is the amplitude of hand displacement, and *t*_*i*_ and *t*_*f*_ are the initial and final instants related to motion.

Mathematically, jerk is the rate of change of acceleration, or the third derivative of position. A high jerk means acceleration is changing rapidly, while a low jerk indicates a smoother, more gradual change in acceleration. To quantify the amount of jerk in hand displacement, the jerk is squared and integrated over the total duration of the movement (Flash and Hogan 1985). According to Hogan and Sternad (2009), this jerk measure depends on the square of the movement length (amplitude of hand displacement) divided by the fifth power of the movement duration, even if the shape of the hand trajectory remains constant. This reliance on movement length and duration is not ideal, so the jerk needs to be normalized (multiplied by the fifth power of movement duration and divided by the square of the movement length). This normalization leads to the final equation presented above.

### 2.5 Trial Exclusion Criteria and Quality Control

Trials were excluded only for a priori protocol or technical reasons, never based on performance outcomes. Sequence compliance required orders were 1‐3‐2 or 3‐1‐2, and a target “hit” was counted when the hand speed was below 0.02 m/s. No dwell was required. Exclusions occurred for wrong orders or missed hits. Trunk stabilization was monitored from thorax–pelvis kinematics using markers at C7, T8, sternal notch, xiphoid (thorax), and bilateral anterior superior iliac spines and S2 (pelvis). Trials were discarded if thorax‐to‐pelvis motion exceeded 20‐mm translation along any axis or 7° rotation at any time during a sequence. The wrist had to remain near the individualized neutral posture; departures greater than ±10° in flexion/extension or radial/ulnar deviation sustained for more than 200‐msec triggered exclusion. We enforced the instructed arm‐movement pattern (“shoulder movement with elbow extension”). Trials showing net elbow flexion across the reach (final elbow angle more flexed than onset) or less than 10° shoulder excursion in the transversal plane were excluded as inconsistent strategies. To uphold the “no pauses” instruction, a full stop between targets was defined as hand speed below 0.02 m/s for at least 1.0 s, and such trials were removed. Finally, motion‐capture failures also led to exclusion if 10% or more of frames for any key marker were missing or if any single gap‐fill exceeded 50 msec.

### 2.6 Statistical Evaluations

Based on our previous report (Contemori et al. 2020), the sample size was estimated by an a priori analysis setting a statistical power of 0.80, an alpha level of 0.05, and a large effect size (0.6) for chi‐square tests (Gpower).

In post hoc evaluations, we accepted significant results with *p* < 0.05 for all statistical tests (except when corrections for multiple comparisons were applied). When we detected outliers and violations of the normality assumptions in the collected samples, as assessed by boxplots and the Shapiro–Wilk test, we used nonparametric tests to compare the data (Statistica, StatSoft; OriginPro, OriginLab Corporation). The Wilcoxon signed‐rank or paired signed‐rank test was used to compare motor performances (movement accuracy, mean speed, peak speed, and NJ) between dependent samples in the basal responses or after interventions within each group (F‐V vs. W‐V). Besides, a Friedman ANOVA test was used to compare motor performance among dependent samples (within groups) over time (preintervention, post‐intervention, and late effect in the retest). In contrast, a two‐sided Mann–Whitney *U* test was used to infer differences between independent samples (i.e., between groups).

The chi‐square and *U* values expressed the significance of the effects. Conover's squared ranks test (using StatsDirect statistical software) verified the homogeneity of the population distribution.

When there were no outliers and data met normality assumptions, Student's *t*‐test or ANOVA designs were performed to evaluate differences among two or multiple samples, respectively. In these cases, Levene's test verified the homogeneity of the variance between populations. If using Student's *t*‐test, the Welch–Satterthwaite correction (Welch *t*‐test) was applied when the homogeneity assumption was violated. Further, Mauchly's sphericity test proved the sphericity assumption, and probabilities were corrected based on Greenhouse–Geisser or Huynh–Feldt epsilon when appropriate.

When significant effects were found, appropriate post hoc tests were applied (Wilcoxon signed‐rank for nonparametric data; Bonferroni's tests for parametric analyses) with Bonferroni correction (accepted significant difference with *p* < 0.017).

Effect sizes were calculated for each test: *r* for nonparametric comparisons, with $r=\frac{\left|z\right|}{\sqrt{N}}.$ (where *Z* is the standard score and *N* is the sample size of each group compared), and partial eta‐squared (*η*_*p*_^2^ = SS_effect_/(SS_effect_ + SS_error_)) for ANOVA.

Data were expressed as mean ± SD or median (Mdn), while graphs showed individual values overlapped to boxplots indicating the interquartile range (box), second quartile (horizontal line within the interquartile range), and whisker plot with outlier range (1.5 box lengths).

## 3 Results

### 3.1 Engagement in Basal Responses

None of the study participants reported muscle fatigue, discomfort, or other side effects during or after vibration or sham intervention.

In the responses recorded before any intervention (Sham or o‐fmv), hereafter referred to as basal responses, aiming movements typically exhibit trajectory inflections due to target undershoot and corrective submovements that were independent in the 3D axes in pre‐, postvibration, and retest for both groups (Exp and Sham) and each condition (F‐V and W‐V) (Figure 2B). As trajectories have inflections, the movement speed exhibits bell‐shaped profiles of velocity and jerk, the rate of change in acceleration along the 3D axes (Figure 2C–D).

**FIGURE 3:**
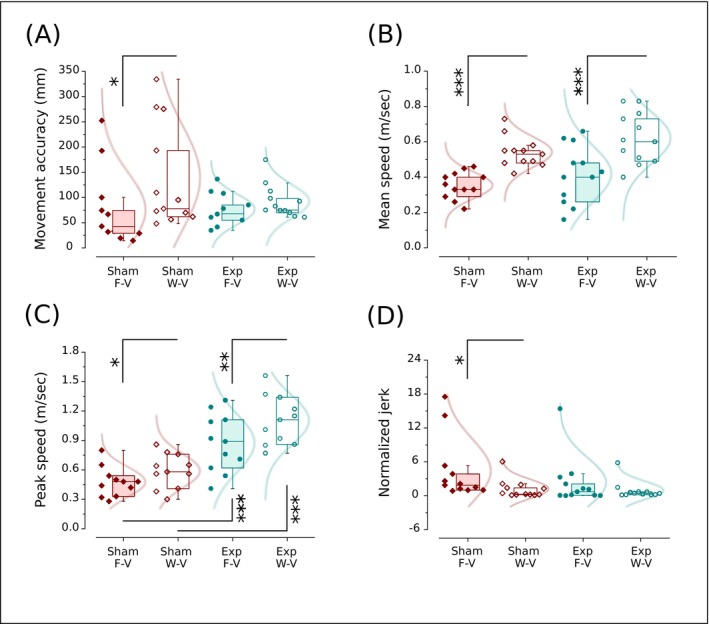
Basal values of movement accuracy (A), mean (B) and peak speed (C), and normalized jerk (D) for the Sham (wine) and Experimental group (Exp, underwent o‐fmv, cyan). Independent engagements are shown between the Sham and Exp (Mann–Whitney *U* test) and paired between conditions F‐V and W‐V (paired *t*‐test or Wilcoxon ranks test). Interquartile range (box) with data overlap (circles or diamonds) and normal distribution fitting (curves), second quartile (horizontal line), and whiskers are represented. F‐V, full vision (visually guided movements); W‐V, without vision (internally guided movements). Asterisk (*) indicates a significant difference (*p < 0.05; **p < 0.01; ***p < 0.001).

However, we found no significant differences in basal responses between Sham and Exp groups for movement accuracy (F‐V, *p* = 0.17; W‐V, *p* = 0.4, Mann–Whitney *U* test), mean speed (F‐V, *p* = 0.23, Welch *t*‐test; W‐V, *p* = 0.16, Mann–Whitney *U* test), and NJ (F‐V, *p* = 0.09; W‐V, *p* = 0.75, Mann–Whitney *U* test), and a significantly higher peak speed in the Exp group than in the Sham group for both F‐V and W‐V conditions (F‐V, *p* < 0.001; W‐V, *p* < 0.001; independent *t*‐test) (Figure 3).

Moreover, comparing basal responses of motor performances between tasks executed in F‐V and W‐V, we found an improved accuracy in the Sham (F‐V versus W‐V*, p* = 0.026, paired *t*‐test) but not in the Exp group (F‐V versus W‐V*, p* = 0.18, *p* = 0.27, paired *t*‐test), a decreased mean speed (Sham: F‐V versus W‐V*, p* < 0.001; Exp Group: F‐V versus W‐V*, p* < 0.001; paired *t*‐test) and peak speed (Sham: F‐V versus W‐V*, p* = 0.014; Exp group: F‐V versus W‐V*, p* = 0.004; paired *t*‐test), and an increased NJ in the Sham (F‐V versus W‐V*, p* = 0.008, paired sign test) but not in the Exp (F‐V versus W‐V*, p =* 0.72, paired sign test) in the F‐V condition (Figure 3).

### 3.2 Evaluations Post‐Interventions

In evaluating changes in motor performance induced by interventions, for the within‐group analysis, we compared values achieved by the investigated variable in the basal task (preintervention) with those performed post‐intervention (Sham or o‐fmv) and retesting. To assess differences in the impact of a condition (W‐V vs. F‐V) within each intervention, we compare responses between W‐T and F‐V in both the Sham and Exp groups using metric values normalized to the basal task.

For the engagements between groups, we compared data calculated as post‐ and late effects (retest) with respect to the preintervention task (basal).

#### 3.2.1 Effect of Interventions on Movement Accuracy

**FIGURE 4:**
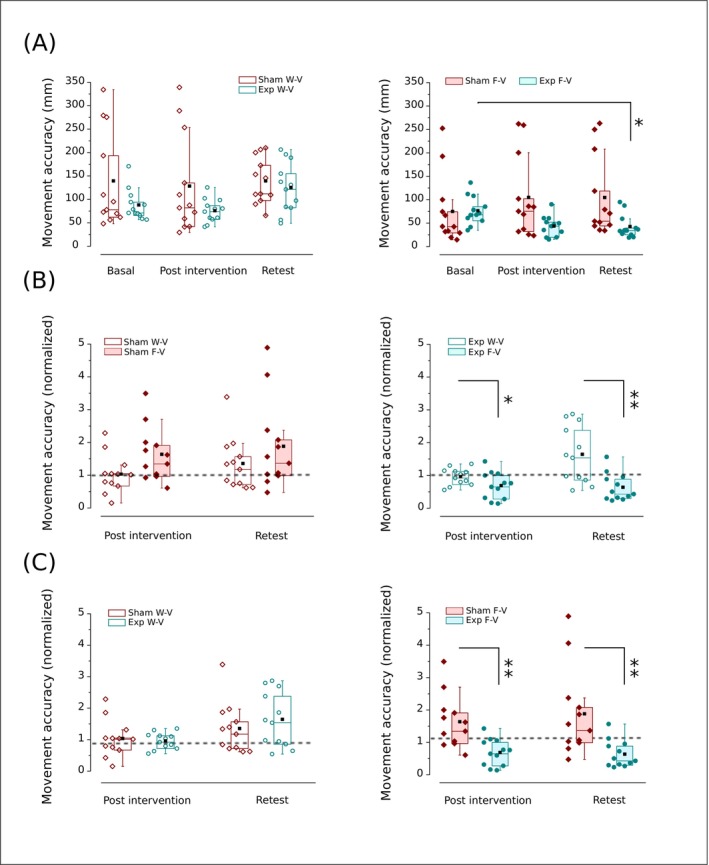
Effect of Sham (wine) or o‐fmv intervention (cyan) on movement accuracy. (A) The within‐group analyses (Sham or Exp) among basal, post‐intervention, and retest (Friedman test with Wilcoxon ranks test) in W‐V (open symbols, top to the left) and F‐V (solid symbols, top to the right). (B) Comparisons between W‐V and F‐V in post‐intervention and retest (signed ranks test) for the Sham (left) and Exp group (right). (C) The between‐groups analyses (Sham vs. Exp) in post‐intervention and retest (Mann–Whitney *U* test) in W‐V (open symbols, bottom to the left) and F‐V (solid symbols, bottom to the right). In (B) and (C), each group's value is normalized to the basal response achieved in the task performed before intervention. The dashed line represents the hypothetical equal value between variables in the normalization. Interquartile range (box) with data overlap (circles or diamonds), second quartile (horizontal line), mean (black square), and whiskers are represented. Exp, Experimental group (underwent o‐fmv); F‐V, full vision (visually guided movements); W‐V, without vision (internally guided movements). Asterisk (*) indicates a significant difference (*p < 0.05; **p < 0.01).

A Friedman test run to determine the movement accuracy trend over time within each group indicated that the difference in accuracy we found among pre‐, post‐intervention, and retest was not statistically significant in the W‐V condition for the Sham (*χ* ^2^ (2, 12) = 0.5, *p* = 0.78) and Exp group (*χ* ^2^ (2, 12) = 1.5, *p* = 0.47) (Figure 4A). In the F‐V condition, differences within groups over time were not significant in the Sham (*χ* ^2^ (2, 12) = 4.7, *p* = 0.097) but significant in the Exp group (*χ* ^2^ (2, 12) = 6.17, *p* = 0.045) (Figure 4A). In the latter case, due to Bonferroni correction, Wilcoxon signed‐rank test post hoc analyses show only a significant accuracy improvement in retest compared to preintervention (*p* = 0.015, *r* = 0.68) and no significant differences in the comparisons involving post‐intervention (*p* > 0.017; Figure 4A).

When analyzing the impact of a condition within each intervention, we found no differences between F‐V and W‐V over time for the Sham (*p* > 0.1, Wilcoxon signed‐rank test; Figure 4B), and higher accuracy in F‐V than W‐V in the post (*p* = 0.03, *r* = 0.62; Wilcoxon signed‐rank test) and late effect (*p* = 0.009, *r* = 0.71, Wilcoxon signed‐rank test) for the Exp group (Figure 4B).

In between‐group analyses performed by a Mann–Whitney *U* test, we found no significant differences between the Sham and Exp group in the W‐V condition for post‐effect (Sham mean rank = 12.75, Exp group mean rank = 12.25, *U* = 75, *p* = 0.88) and late effect (Sham mean rank = 11.42, Exp group mean rank = 13.58, *U* = 59, *p* = 0.47) (Figure 4C) and significantly better accuracy in the Exp group than the Sham in the F‐V condition for post effect (Sham mean rank = 16.83, Exp group mean rank = 8.16, *U* = 124, *p* = 0.002, *r* = 0.85) and late effect (Sham mean rank = 17, Exp group mean rank = 8, *U* = 126, *p* = 0.001, *r* = 0.89) (Figure 4C).

#### 3.2.2 Effect of Interventions on the Mean Speed of Movement

**FIGURE 5:**
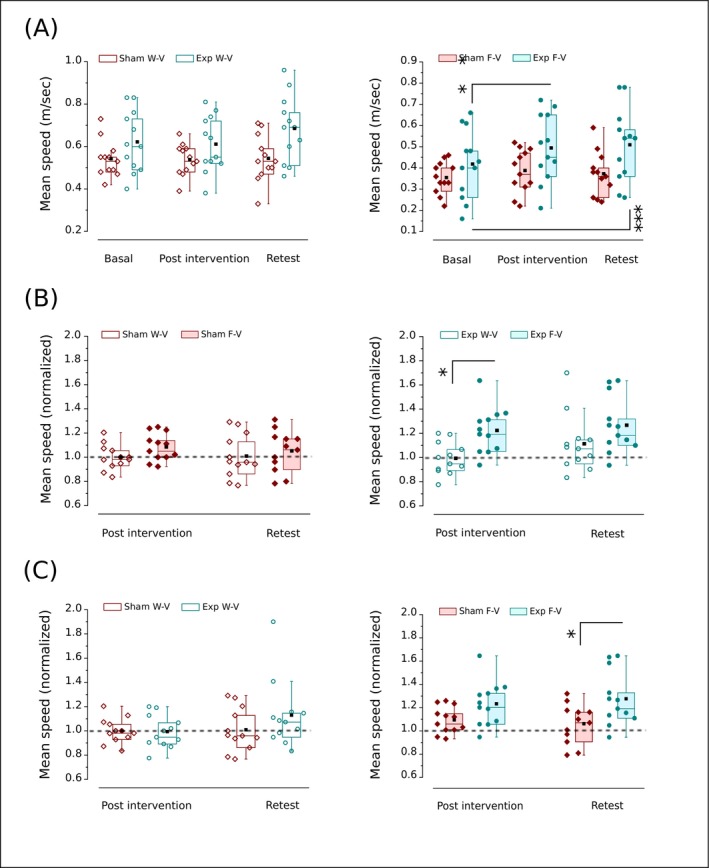
Effect of Sham (wine) or o‐fmv intervention (cyan) on the mean speed of the movement. (A) The within‐group analyses (Sham or Exp) among basal, post‐intervention, and retest (one‐way repeated measures ANOVA with Bonferroni) in W‐V (open symbols, top to the left) and F‐V (solid symbols, top to the right). (B) Comparisons between W‐V and F‐V in post‐intervention and retest (signed ranks test) for the Sham (left) and Exp group (right). (C) The between‐group analyses (Sham vs. Exp) in post‐intervention and retest (Mann–Whitney *U* test) in W‐V (open symbols, bottom to the left) and F‐V (solid symbols, bottom to the right). In (B) and (C), each group's value is normalized to the basal response achieved in the task performed before intervention. The dashed line represents the hypothetical equal value between variables in the normalization. Interquartile range (box) with data overlap (circles or diamonds), second quartile (horizontal line), mean (black square), and whiskers are represented. Exp, Experimental group (underwent o‐fmv); F‐V, full vision (visually guided movements); W‐V, without vision (internally guided movements). Asterisk (*) indicates a significant difference (*p < 0.05; **p < 0.01; ***p < 0.001).

In the W‐V condition, we used a one‐way repeated measures ANOVA to examine the mean movement speed within each group over time. Although there were outliers, the normality and shape of the distribution do indeed suggest that using a parametric test for statistical comparisons is acceptable (Figure 5A). We found no significant difference in the mean speed of movement in the Sham group (*F*(2,22) = 0.03, *p* = 0.96) or the Exp group (*F*(2,22) = 2.68, *p* = 0.09) (Figure 5A). On the other hand, the analyses of mean speed within groups over time in the F‐V condition run by a one‐way repeated measures ANOVA did not elicit significant differences in the Sham among pre‐, post‐intervention, and retest (*F*(2,22) = 1.69, *p* = 0.21) but significant in the Exp group (*F*(2,22) = 12.77, *p* < 0.001; *η*_*p*_^2^ = 0.54) (Figure 5A). Consistently, in the Exp group, the Bonferroni post hoc test shows that the mean speed significantly increases from basal to post‐intervention (*p* = 0.002) and from basal to retest (*p* < 0.001) (Figure 5A). When analyzing the impact of a condition within each intervention, we found no differences between F‐V and W‐V over time for the Sham (*p* > 0.1, Wilcoxon signed‐rank test; Figure 5B), and a significantly higher mean speed increase in F‐V than W‐V in the post (*p* = 0.016, *r* = 0.68; Wilcoxon signed‐rank test) and not significant in late effect (*p* = 0.051, *r* = 0.62, Wilcoxon signed‐rank test) for the Exp group (Figure 5B).

Moreover, a Mann–Whitney *U* test conducted in between‐group analyses determined no significant difference between the Sham and Exp groups in the W‐V condition (post effect: Sham mean rank = 12.96, Exp group mean rank = 12.04, *U* = 77.5, *p* = 0.77; late effect: Sham mean rank = 10.91, Exp group mean rank = 14.08, *U* = 53, *p* = 0.26) (Figure 5C). Besides, while the mean speed between the Exp group (mean rank = 15.25) and Sham (mean rank = 9.75) showed no significant difference in post effect (*U* = 39, *p* = 0.054), it was significantly higher in the Exp group (mean rank = 15.51) than in the Sham (mean rank = 9.3) in the retest (*U* = 36, *p* = 0.03, *r* = 0.74) for F‐V condition (Figure 5C).

#### 3.2.3 Effect of Interventions on the Peak Speed of Movement

**FIGURE 6:**
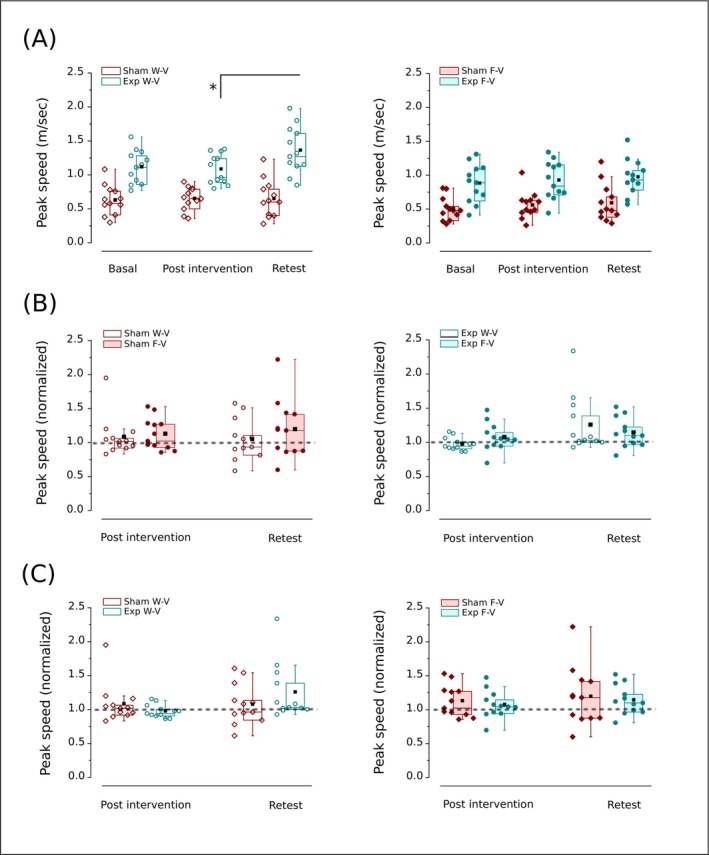
Effect of Sham (wine) or o‐fmv intervention (cyan) on the peak speed of the movement. (A) The within‐group analyses among basal, post‐intervention (Sham or Exp), and retest in W‐V (open symbols, top to the left; one‐way repeated measures ANOVA with Bonferroni) and F‐V (solid symbols, top to the right; Friedman test). (B) Comparisons between W‐V and F‐V in post‐intervention and retest (signed ranks test) for the Sham (left) and Exp group (right). (C) The between‐group analyses (Sham vs. Exp) in post‐intervention and retest (Mann–Whitney *U* test) in W‐V (open symbols, bottom to the left) and F‐V (solid symbols, bottom to the right). In (B) and (C), each group's value is normalized to the basal response achieved in the task performed before intervention. The dashed line represents the hypothetical equal value between variables in the normalization. Interquartile range (box) with data overlap (circles or diamonds), second quartile (horizontal line), mean (black square), and whiskers are represented. Exp, Experimental group (underwent o‐fmv); F‐V, full vision (visually guided movements); W‐V, without vision (internally guided movements). Asterisk (*) indicates a significant difference (*p < 0.05).

The analysis of the peak speed of the movement within groups over time run by a one‐way repeated measures ANOVA detected no significant differences in W‐V conditions for the Sham among pre‐, post‐intervention and retest (*F*(2,22) = 0.13, *p* = 0.87) and a significant difference for the Exp group (*F*(1.14, 12.56) = 6.80, *p* = 0.018, *η*_*p*_^2^ = 0.41) (Figure 6A). Bonferroni post hoc test conducted for the Exp group shows no significant changes in peak speed from pre‐ to postvibration (*p* = 0.91), and from previbration to retest due to Bonferroni correction (*p* = 0.019), while a significantly higher peak speed of retest than postvibration (*p* = 0.008) (Figure 6A).

On the other hand, a Friedman test was conducted for statistical analysis in F‐V, as outliers were present in the data collected under this condition for the within‐group design (Figure 6A). Neither the Sham (*χ* ^2^ (2, 12) = 0.67, *p* = 0.72) nor the Exp group (*χ* ^2^ (2, 12) = 2.16, *p* = 0.34) showed significant differences (Figure 6A). When analyzing the impact of a condition within each intervention, we found no significant difference between F‐V and W‐V in the Sham (*p* > 0.1, Wilcoxon signed‐rank test) (Figure 6B) nor in the Exp (*p* > 0.2, Wilcoxon signed‐rank test) (Figure 6B).

In between‐group analyses, using a Mann–Whitney *U* test, we found no significant difference between the Sham and Exp groups in both W‐V (post effect: Sham mean rank = 14, Exp group mean rank = 11, *U* = 90, *p* = 0.31; late effect: Sham mean rank = 10.5, Exp group mean rank = 14.5, *U* = 48, *p* = 0.17) (Figure 6C) and F‐V condition (post effect: Sham mean rank = 12.83, Exp group mean rank = 12.2, *U* = 76, *p* = 0.83; late effect: Sham mean rank = 12.58, Exp group mean rank = 12.42, *U* = 73, *p* = 0.96) (Figure 6C).

#### 3.2.4 Effect of Interventions on NJ

**FIGURE 7:**
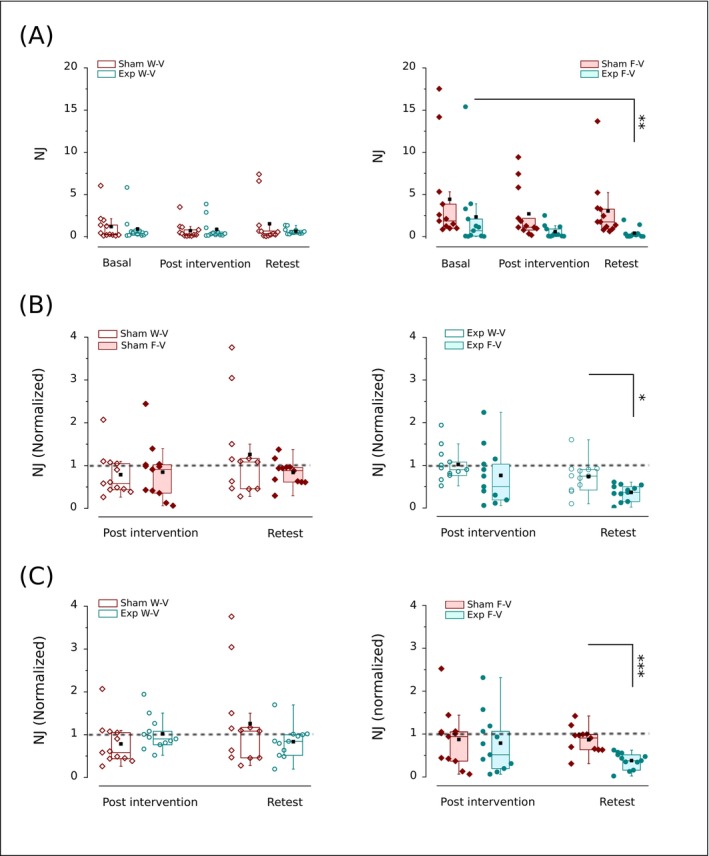
Effect of Sham (wine) or o‐fmv intervention (cyan) on normalized jerk (NJ). (A) The shown the within‐group analyses among basal, post‐intervention (Sham or Exp), and retest (Friedman test with Wilcoxon ranks test) in W‐V (open symbols, top to the left) and F‐V (solid symbols, top to the right). Values have to be timed by 10^6^. (B) Comparisons between W‐V and F‐V in post‐intervention and retest (signed ranks test) for the Sham (left) and Exp group (right). (C) The between‐group analyses (Sham vs. Exp) in post‐intervention and retest (Mann–Whitney *U* test) in W‐V (open symbols, bottom to the left) and F‐V (solid symbols, bottom to the right). In (B) and (C), each group's value is normalized to the basal response achieved in the task performed before intervention. The dashed line represents the hypothetical equal value between variables in the normalization. Interquartile range (box) with data overlap (circles or diamonds), second quartile (horizontal line), mean (black square), and whiskers are represented. Exp, Experimental group (underwent o‐fmv); F‐V, full vision (visually guided movements); W‐V, without vision (internally guided movements). Asterisk (*) indicates a significant difference (**p < 0.01; ***p < 0.001).

The within‐groups analyses of NJ over time conducted by a Friedman Anova test show no significant changes in the W‐V condition for the Sham (*χ* ^2^ (2, 12) = 3.5, *p* = 0.17) but significant changes for the Exp group (*χ* ^2^ (2, 12) = 7.18, *p* = 0.028) (Figure 7A). However, the Wilcoxon signed‐rank test post hoc analysis run for the Exp group does not elicit a significant difference between the values of NJ in pre‐, postvibration, and retest due to the Bonferroni correction (accepted significant difference with *p* < 0.017) (Figure 7A). Similarly, a Friedman test used for the analyses of NJ over time in the F‐V condition shows no significant changes in the Sham (*χ* ^2^ (2, 12) = 3.6, *p* = 0.16) and significant changes in the Exp group (*χ* ^2^ (2, 12) = 12.17, *p* = 0.0023) (Figure 7A). In the latter case, Wilcoxon signed‐rank test post hoc analyses revealed a significantly lower value of NJ in the retest than in the previbration condition (*p* = 0.0015; *r* = 0.91), while no differences were found in the other comparisons (*p* > 0.017) (Figure 7A). When analysing the impact of a condition within each intervention, we found no differences between F‐V and W‐V over time for the Sham (*p* > 0.5, Wilcoxon signed‐rank test; Figure 7B), and no significant difference in NJ between F‐V and W‐V in the post (*p* = 0.47; Wilcoxon signed‐rank test) and a significantly lower NJ in F‐V than W‐V in the late effect (*p* = 0.027, *r* = 0.62, Wilcoxon signed‐rank test) for the Exp group (Figure 7B).

Moreover, a Mann–Whitney *U* test performed to study NJ values between‐group detected no significant difference between the Sham and Exp groups in W‐V (post effect: Sham mean rank = 10.25, Exp group mean rank = 14.75, *U* = 45, *p* = 0.13; late effect: Sham mean rank = 14.33, Exp group mean rank = 10.67, *U* = 93, *p* = 0.24) (Figure 7C). However, in F‐V, Mann–Whitney *U* tests showed no significant difference between the Sham (mean rank = 13.17) and Exp group (mean rank = 11.83) in post effect (*U* = 80, *p* = 0.66) but a significantly lower NJ value in the Exp group (mean rank = 7.25) than in the Sham (mean rank = 17.76) in retest (*U* = 135, *p* < 0.001; *r* = 0.96) (Figure 7C).

## 4 Discussion

In this investigation with healthy participants, we found that the o‐fmv has a consistent and long‐lasting impact on upper limb goal‐directed aiming when visual information is available in real time, whereas its effect is minimal when movements rely on memorized visual information. Indeed, the study's results indicate that the o‐fmv applied before performing motor tasks improves motor performance in aiming movements when motion planning, programming, and execution are accomplished with visual feedback. The improvement in motor performance includes accuracy, speed, and smoothness of movement, and this enhancement began immediately and is most notable 1 week after the o‐fmv application, as per the follow‐up period we used. Conversely, when planning but not the programming and execution phases of the movement are under vision (i.e., internally controlled movements toward targets in memorized positions), the o‐fmv only slightly affects the upper limb kinematics over time. Our findings highlight the basal features of aiming movements in processing sensorimotor information, underscore proprioception's crucial contribution during the computational stages of planning and execution, and indicate that the o‐fmv may improve these processes primarily when vision is engaged during ongoing motion.

### 4.1 Basal Motor Behavior: Movement Accuracy and Kinematics

A common feature among all aiming movements studied is the independent control of each of the 3D coordinates (Figure 2). Besides, participants frequently undershot the target and performed corrective submovements to reach the target in all basal setups (Figure 2). They might combine proprioception with visual information available in real time (i.e., visually guided movements in the F–V condition) or with an internal representation of the target–hand positions when vision is occluded, relying on memorized visual information (i.e., internally or memory‐guided movements in the W–V condition) to control and correct goal‐directed movements. (Figure 2). At the same time, overshoots were rarely observed. Undershooting the target probably represents a conservative approach to avoiding cost movement errors that occur from correcting overshooting (Lyons et al. 2006).

Moreover, the results indicate that internally guided movements may be less accurate and more variable than those guided visually (Figure 3). Indeed, in basal responses, vision improved accuracy and reduced variability in the Sham group. In contrast, in the Exp group, there is only a tendency to improve accuracy in vision that does not reach statistical significance (Figure 3). On the other hand, visual feedback always showed a significant influence on the speed of the movement, slowing it (Figures 2D and 3) and revealing that speed of movement is more subjected than accuracy to task‐dependence control mechanisms. Although with a general trend to be more variable in vision, movement smoothness was similar when visually or internally guided in the Exp group but not in the Sham (Figure 3). In the latter case, the smoothness worsens in the presence of visual feedback, as shown by the increase in NJ (Figure 3).

The effect of vision in basal responses, especially in slowing speed and worsening smoothness, could be unexpected (Hansen et al. 2006). However, while we detected robust differences between F‐V and W‐V in movement mean speed, these differences became much less consistent for peak speed. This trend was evident in some participants, whose performances showed a low mean with relatively high peak speed in F‐V, indicating irregularity in motor task execution that possibly caused an increase in jerk (Figure 3).

We speculate that participants plan for the worst case scenario under conditions of visual feedback uncertainty, as in our experimental design with randomly arranged trials of either visually or internally guided movement, motion resembling the no‐vision approach for all conditions, primarily relying on an advanced‐built spatial plan (Khan et al. 2006). In this schema, participants may adopt an initial open loop plan of limb control to get to the target area and minimize the movement time cost in the absence of visual processing. As a consequence, bell‐shaped velocity profiles are expected (Figure 2). When vision was available, however, a slower mean speed than without visual feedback would reflect the time cost needed to prepare for transitioning to an online movement control strategy and the visual processing to meet the task's accuracy demands (Figures 2D–3) (Schmidt et al. 1979). In that case, movements would result in a skewed velocity profile with more time spent after peak velocity (Figure 2D) (Khan et al. 2006). Consequently, visually guided movements could increase the time spent aiming at visual targets (Sarlegna et al. 2006; Krigolson and Heath 2004), reducing the mean more than peak speed (Figures 2D and 3).

### 4.2 Immediate and Enduring Aftereffects of Interventions on Accuracy and Kinematics of Aiming

We found that the o‐fmv paradigm was highly effective in improving the kinematics and accuracy of participants' aiming movements when, from planning to execution, every phase of the movement was performed with the visual feedback of the upper limb, targets, and backgrounds.

In internally guided movement performed after the intervention, the Sham (the group that underwent fake vibration) and Exp (the group that underwent o‐fmv) do not significantly change in accuracy from basal to retest (Figure 4A). Additionally, they follow a similar trend in accuracy over time, probably reflecting the “training” effect of trial sequences. In visually guided movements, for the Sham group, there is a slight decrease in accuracy from basal to long‐term (Figure 4A). Conversely, for the Exp group, accuracy immediately shows a trend to increase, which becomes significant and less variable in the long term (retest) (Figure 4A). While the Sham group did not show a significant difference over time between visually and internally guided aiming, the Experimental group exhibited an immediate disparity between the two setup conditions in guiding aiming, which consolidated in the long term (Figure 4B). Besides, no differences over time were detected between the two groups for internally guided movements (Figure 4C). However, the Exp group shows an immediate increase in accuracy compared to the Sham in visually guided movement, which is maintained in the long term (Figure 4C).

For mean speed in internally guided movements, we detected changes over time in neither the Sham nor the Exp group (Figure 5A). In contrast, for visually guided movements, we found a robust increase from basal, through post‐intervention, to long‐term in the Exp group, without significant changes in the Sham group (Figure 5A). As a result of these trends, o‐fmv nulls over time, the basal bias for mean speed is higher internally than visually guided movements (Figures 3B and 5A). Similarly to accuracy, the Sham does not show a significant difference in speed over time between visually and internally guided aiming. In the Exp group, however, there is an immediate difference between visually and internally guided aiming, which is maintained in the long term (Figure 5B). At the same time, we did not find differences in movement speed over time between the two groups for internally guided movements, while the Exp group speed has an immediate trend of being higher than the Sham in visually guided movement, which becomes significant in the long term (Figure 5C).

Moreover, we considered the rate of acceleration changes to express movement regularity in the follow‐up period of observation (Balasubramanian et al. 2012). When kinematics vary most regularly over time, a maximal smoothness of movement is achieved (Balasubramanian et al. 2015). Thus, jerk cost may quantify smoothness by calculating the NJ, with minimal jerk reflecting maximal smoothness (Roren et al. 2021; Flash and Hogan 1985). Our results show a long‐term o‐fmv effect (Exp group) in ameliorating motion regularity for visually guided aiming, with the increase in movement smoothness (decrease in NJ) reaching significance later in the retest (Figure 7A). This increase was parallel to the decrease in variability (Figure 7A). Conversely, the smoothness does not change over time in the Exp group for internally guided movements. Therefore, o‐fmv inverts the basal trend for smoothness to be better in internally than visually guided movements (Figures 3D and 7C). Moreover, there are no significant changes in smoothness in Sham for both internally and visually guided movements (Figure 7B). As a result, in the long term and only for visually guided movements, the Exp group shows significantly higher smoothness increase compared to the Sham (Figure 7C).

Movement speed is critical for accurate execution, and increasing speed without practice is costly due to possible accuracy impairment, and energetic and smoothness costs are expenditures for error corrections (Elliott et al. 2017; Lyons et al. 2006; Schmidt et al. 1979). Usually, performers adopt an approach to motion avoiding cost movement errors and enhancing speed, accuracy, and smoothness that happens mostly after extensive practice, probably reflecting changes in sensorimotor domains that are related to improved motor execution (Elliott et al. 2017; Hansen et al. 2006; Balasubramanian et al. 2015; Sejnowski 1998; Schneider and Zernicke 1989). Besides, improvements in kinematics and accuracy occurring trial‐by‐trial may reflect refinement in predictive and impedance mechanisms of movement control as an adaptive response to training (Scheidt and Ghez 2007).

It is worth noting that, under our experimental conditions, practicing the motor task per se, from basal responses to post‐intervention and retesting, did not significantly improve movement accuracy, speed, or smoothness. Indeed, the Sham shows significant changes in accuracy and kinematics, both with and without vision, similar to what the Exp group mostly does in the absence of vision during task performance (Figures 4, 5, 6, 7).

Therefore, our finding that movement accuracy and kinematics begin improving immediately after o‐fmv in visually guided movement (F‐V condition) indicates that performers are more suitable for enhancing their motor control under these circumstances. Additionally, with minimal practice in the experimental task trials, performers in the Exp group improved their global movement performance, similar to what is typically observed after conventional, more extensive training. In fact, after o‐fmv, accuracy and kinematics enhancement in the F‐V condition show a robust trend of progressively increasing from immediate to long‐term (Figures 4, 5 and 7). Hence, our findings imply that the o‐fmv may enhance motor control mechanisms and favor learning‐like phenomena over time.

Our results are allied with previous data indicating that muscle vibration may induce a gradual and long‐term effect on sensory–motor features (Brunetti et al. 2012; Filippi et al. 2009; Contemori et al. 2020; Pettorossi et al. 2015). The phenomenon could be related to the time the neuromuscular system needs after optimized stimulations to gradually determine changes, possibly underlying the modulation of various features of movement performance (Marconi et al. 2008; Ridding et al. 2001; Rosenkranz and Rothwell 2012; Pfenninger et al. 2023).

Overall, the data confirm the potential of focal muscle vibration in inducing aftereffects on the motor domain when it meets optimal requirements, as previously observed (Contemori et al. 2020). Additionally, assuming they alter proprioceptive information processing, optimized stimulations should act under favorable circumstances to achieve maximal effectiveness over time, as we found for o‐fmv, which primarily impacts movement coordination when proprioception can be combined with visual information during ongoing movement.

### 4.3 Possible Action Mechanisms of the O‐fmv in Modulating Goal‐Directed Aiming

A reasonable assumption is that our stimulation paradigm may influence the visual–proprioceptive network that controls goal‐directed aiming and visuomotor coordination (Butler et al. 2004; Heath and Westwood 2003).

Perhaps o‐fmv enhances the optimization of movement kinematics and accuracy, which depends on improving sensitive and motor processes (Khan et al. 1998). It is admissible that such enhancement could be mediated by a more efficient central representation for goal behavior and movement‐associated processes due to o‐fmv impacting proprioception and visual–proprioceptive interaction. Consistently, muscle vibration used in the o‐fmv is a repetitive and potent stimulator of muscle spindles' primary endings and other musculotendinous, cutaneous, and articular proprioceptors, such as secondary spindle endings, Golgi tendon organs, Pacinian and Meissner corpuscles, and even slowly adapting cutaneous low threshold receptors (Strzalkowski et al. 2017; Fallon and Macefield 2007; Roll et al. 1989; Ribot‐Ciscar et al. 1996; Burke et al. 1976). The broad and intense afferent recruitment, combined with motor activation due to the o‐fmv (see next section), may lead to sensitization‐like phenomena, favoring changes in the somatosensory circuitry and possibly promoting the enhancement of proprioceptive information processing. Other systems have shown a similar phenomenon following repetitive stimulation (Clapp et al. 2012; Seitz and Dinse 2007).

Additionally, the impact of o‐fmv on proprioception may be preferred by affecting the fusimotor drive to ongoing movements (Hospod et al. 2007; Ribot‐Ciscar et al. 2009; Pope and DeFreitas 2015). As a result, spindle sensitivity may be optimized to better suit the needs of muscles and joints for successful action execution (Lan and He 2012). Accordingly, under o‐fmv impact, the proprioceptive network could increase its capability to encode positional and motion signals (Filippi et al. 2023; Ribot‐Ciscar et al. 2009). The consequence for the brain could be more efficient spatial and dynamic information processing, improving the motor plan and converting it into more effective muscle activations for robust movement coordination. A better movement representation and motor plan conversion can lead performers to exploit predictive and joint impedance control mechanisms, ameliorating kinematics, smoothness, and final position error (Gordon et al. 1995; Gribble et al. 2003; Scheidt and Ghez 2007).

On the other hand, the effective control of goal‐directed aiming is closely tied to integrating visual–spatial and proprioceptive signals before (action planning and programming period) and during action (Medendorp et al. 2005; Sober and Sabes 2005; Scheidt et al. 2005; Elliott 1988; Khan et al. 2006). Given this, if it reorganizes the neuronal processing of proprioceptive information, it is unsurprising that o‐fmv impacts movement performance mainly during motion execution in vision, as evidenced in our study. Indeed, if vision is not accessible immediately prior to movement decision (action programming) and during task execution, proprioception cannot be combined with any updated visual information in this period, and proprioceptive signals cannot exert real‐time refinement and unfold an updated visual–spatial program (Binsted et al. 2006; Goodale et al. 2004). Movement corrections are still possible based on proprioception, matching internal visually outdated references (Figures 2, 3) (Sarlegna et al. 2006; Heath and Westwood 2003), although the capability to improve accuracy and kinematics can deteriorate (Figures 3, 4, 5 and 7).

We speculate that, at the higher central level, o‐fmv could influence the visual stream pathways involved in real‐time visuomotor coordination, where sensory signals trigger sensorimotor events that initiate goal‐directed actions (Siegel et al. 2015; Medendorp et al. 2005; Heath 2005). Supporting this speculation is the fact that visual stream pathways combine visual and proprioceptive flows encoding target, hand position, and limb dynamics and comprehend areas with reaching neurons representing movement in a shoulder‐centric frame of reference where different neuronal subpopulations selectively encode each of the 3D coordinates (Battaglia‐Mayer et al. 2000; Battaglia‐Mayer et al. 2005). The dorsal stream of pathways is active during the preparatory phase and when movement is ongoing, and it is responsible for the online control of movement amplitude, direction, and speed in goal‐directed actions. Nevertheless, control of manual aiming driven by visual–proprioceptive processing could require information even from the ventral visual stream pathway (Binsted et al. 2006). Thus, more generally, in our model, the main o‐fmv impact appears to be confined to aiming movements initiated by the activation of a route using vision in real‐time, as the o‐fmv effect on kinematics and accuracy in the experimental F‐V condition suggests. Consistently, o‐fmv only slightly affects motion kinematics of internally driven aiming, as we found in our experimental W‐V condition, which still relies on proprioception but does not require up‐to‐date data from visual pathways for motor plan unfolding.

Moreover, while the F‐V and W‐V conditions are similar in integrating visual–proprioceptive signals for planning, they differ in movement programming and execution, particularly in the use of real‐time (F‐V) or memorized (W‐V) visual information. Thus, from a neurophysiological perspective, it is reasonable to assume that o‐fmv primarily impacts information processing in movement programming and execution via a pathway activation route that relies on real‐time visual information. For goal‐directed movement, programming is the phase of motor control in which an already built spatial plan is transformed into a motor program (Goodale et al. 2004). The more refined the transformation, the more accurate the movement program and execution should be.

Consistently, somatosensory stimulation, proprioceptive‐based training, and volitional muscle activation may enhance the neuronal computational capability of proprioception and possibly mediate changes within the sensorimotor cortices that are part of the dorsal stream (Aman et al. 2014; Ridding et al. 2001; Rosenkranz and Rothwell 2004; Giangrande et al. 2024). In particular, focal muscle vibration may be a potent modulator of cortical structures usually activated during proprioceptive stimulation and related to motor practice (Souron, Besson, Millet, et al. 2017; Classen et al. 1998; Forner‐Cordero et al. 2008; Rosenkranz and Rothwell 2003; Vahdat et al. 2011; Amiez et al. 2024; Lapole et al. 2015). When administered at high‐frequency, focal muscle vibration may promote plastic changes in the primary motor cortex related to the vibrated, neighboring, and antagonist muscles associated with remodeling intracortical and cortical reciprocal inhibition that can persist over time after vibration (Rosenkranz and Rothwell 2012; Marconi et al. 2008; Li et al. 2019; Steyvers et al. 2003). However, we do not know which modification in the sensorimotor system exactly underlies the improvement in movement control we observed after the o‐fmv. Phenomena at the spinal and muscular level may take part in the improvement as muscle vibration could influence spinal pathways and induce functional changes in plasticity at the spinal cord (Rocchi et al. 2018; Souron, Besson, Millet, et al. 2017; Pfenninger et al. 2023; Amiez et al. 2024) and potentially even structural alteration in the vibrated muscles (Proske and Gandevia 2012; Marin et al. 2013). However, while changes in the mechanical muscle fibers' properties after vibration are possible, as in the case of thixotropic remodeling, it is unlikely that they may mediate the lasting aftereffect of the o‐fmv (White and Proske 2009).

Nevertheless, to our knowledge, very little is known about focal vibration effects on remodeling the macroscopical arrangement of muscle fibers. Additional experiments are essential to improve our understanding of the mechanisms behind o‐fmv effects.

### 4.4 The Optimized Stimulation Paradigm and Its Significance for Behavioral Modulation

We utilize focal muscle vibrations as an essential stimulus component to promote long‐lasting changes in sensorimotor behavior (Contemori et al. 2020; Pettorossi et al. 2015; Filippi et al. 2009). One main goal has been determining a stimulation paradigm via inflow–outflow manipulation to enhance the sensorimotor function without extensive and explicit task training (Beste and Dinse 2013; Seitz and Dinse 2007). This approach is quite different from the conventional one, which uses motor learning principles to achieve improvement by practicing (Khan et al. 1998; Scheidt and Ghez 2007). Our data point to optimal stimulation in promoting an associative convergence between multisensory inflow and motor activation, possibly facilitating Hebbian cooperative phenomena in central networks responsible for sensorimotor processing (Pleger et al. 2003; Pettorossi et al. 2015; Giangrande et al. 2024; Beste and Dinse 2013). Optimal stimulation should possess permissive factors per se, including specific temporal stimulation propriety, size, and spatial relevance of the stimulated area, effective involvement of stimulated effectors for the explored function, number, duration, and sequence of applied stimuli (Contemori et al. 2020; Filippi et al. 2023; Seitz and Dinse 2007). For instance, as the vibratory stimulation at 100 Hz we used in our model, a high frequency of sinusoidal mechanical oscillation may be followed by primary spindle endings and cutaneous Meissner and Pacinian corpuscles that fire into the proprioceptive network at a frequency capable of inducing synaptic plasticity in brain slice preparations (Strzalkowski et al. 2017; Fallon and Macefield 2007; Roll et al. 1989; Clapp et al. 2012). Besides, combining such high‐frequency vibration with intentional muscle contraction during stimulation may facilitate spindle activation on the one hand and recruit central motor drives in association with an increased proprioceptive inflow on the other. The idea is to combine potentially optimal factors to facilitate spatiotemporal cooperative phenomena and possibly promote plastic rearrangements in the activated neural circuits, resulting in enhanced neurocognitive processes that may shape behavior (Beste and Dinse 2013; Contemori et al. 2020; Clapp et al. 2012). Moreover, we found that the o‐fmv specifically affects aiming movements in the F‐V condition, representing an additional favorable circumstance in network route convergence, where proprioception can be combined with vision (see previous section) during motion preparation and ongoing movements (Battaglia‐Mayer 2019). Generally speaking, it is admissible that optimized stimulation may alter central information processing, but passing the neuronal signal's responsiveness threshold to induce behavioral effects requires combining more complex and favorable circumstances.

However, although it may result in similar changes to behavior as training, the optimized stimulation would need little or no practice and could help acquire or improve skills related to the activated domains.

From a neuroscience perspective, optimized stimulation can potentially operate in the area of repair and reorganization to help restore sensorimotor abilities on the one hand and cognition and regular learning to enhance function or compensate for a deficit on the other.

### 4.5 Limitations

The current study still has several limitations to consider. First, we made some assumptions about the proprioceptive pathways activated by the o‐fmv, but our approach, although supported by substantial literature, only indirectly investigated this aspect. Additionally, we cannot distinguish between the relevance of visual signals in influencing movement execution and the earlier phases of motion preparation, nor the time course at which they can be held available to match with proprioception for effective visual–proprioceptive integration. Similarly, our approach cannot directly detect neuroplasticity or network changes at a structural level. Thus, further research is required to explore in more detail the process triggered by o‐fmv and the factors modulated, resulting in alterations to motor skills.

Other limitations could be related to the use of a quite broad age range of participants in the study, the lack of separation between right‐ and left‐handed individuals, and between males and females. All of these factors may introduce variability and bias the results. Therefore, caution is necessary to avoid overgeneralizing the results. Besides, participants' interpretation of the instruction to perform motor tasks as quickly and accurately as possible may also be a limiting factor. However, a critical point of the study is investigating the relationship between the speed, accuracy, and smoothness of aiming movements. The request to be quick and accurate in task execution allows participants to find their optimal compromise between these movement features.

Regarding the number of participants, we estimated the sample size through a prior power analysis to maintain the typical minimum probability acceptable for both Type I and Type II statistical errors. Post hoc evaluations revealed a similar or larger effect size than those used in a priori assumptions, indicating that the number of participants in our investigation is adequate (Serdar et al. 2021).

## 5 Conclusion

In this investigation with healthy participants, we found that the o‐fmv has a consistent and long‐lasting impact on upper limb goal‐directed aiming when visual information is available in real time, whereas its effect is minimal when movements rely on memorized visual information.

The primary effect involves enhanced upper limb motor performance in goal‐directed movements when tasks are prepared and executed in a moment‐to‐moment manner, utilizing real‐time visual information. The improvement involves enhanced kinematics and ameliorated accuracy, which began immediately and continues to increase 1 week after the o‐fmv. Furthermore, in visually guided movements, o‐fmv promotes changes in motion coordination with minimal practice, resembling the outcomes achievable through more conventional approaches that require extensive training.

The o‐fmv exerts its effect, probably influencing the visual–proprioceptive interaction when sensorimotor transformation mechanisms serving motor output rely on an online visual mode for movement control. Indeed, our data indicate that o‐fmv may enhance the brain's processing of proprioceptive information, supporting processing during the computational stages of planning and during execution for effective motion coordination, particularly when movements are initiated through pathways that rely on real‐time visual information. The latter is a function attributed to the brain's visual stream circuitry, which controls online visuomotor transformations, implying that o‐fmv may induce a long‐term influence in that pathway. Finally, since the o‐fmv holds promise in promoting changes that generate efficient volitional movement coordination, we suggest considering it to develop intervention strategies that improve motor skills, restore, or even compensate for motor cognition impairments or sensorimotor disorders.

## Author Contributions

**Roberto Panichi:** conceptualization, data curation, formal analysis, investigation, methodology, project administration, supervision, validation, visualization, writing – original draft, writing – review and editing. **Samuele Contemori:** data curation, formal analysis, investigation, methodology, software, writing – review and editing. **Jacqueline A. Sullivan:** data curation, validation, writing – review and editing. **Andrea Calandra:** investigation, software, writing – review and editing. **Cristina V. Dieni:** conceptualization, data curation, visualization, writing – review and editing. **Andrea Biscarini:** conceptualization, data curation, formal analysis, methodology, resources, software, writing – review and editing.

## Conflicts of Interest

The authors declare no conflicts of interest.

## Data Availability

Data supporting the findings of this study are available from the corresponding author upon reasonable request.
